# A recurrent multimodal sparse transformer framework for gastrointestinal disease classification

**DOI:** 10.1038/s41598-025-08897-0

**Published:** 2025-07-07

**Authors:** V. Sharmila, S. Geetha

**Affiliations:** School of Computer Science and Engineering, Vellore Institute of Technology, Chennai, 600127 India

**Keywords:** Gastrointestinal disease classification, Multimodal feature fusion, Bio-RoBERTa, Sparse transformer network, Cross-attention mechanism, Gastrointestinal models, Computational models

## Abstract

Accurate and early diagnosis of gastrointestinal (GI) tract diseases is essential for effective treatment planning and improved patient outcomes. However, existing diagnostic frameworks often face limitations due to modality imbalance, feature redundancy, and cross-modal inconsistencies, particularly when dealing with heterogeneous data such as medical text and endoscopic images. To bridge these gaps, this study proposes a novel recurrent multimodal principal gradient K-proximal sparse transformer (RMP-GKPS-transformer) framework for comprehensive GI disease classification. The approach integrates clinical text and WCE images using a robust multi-modal fusion strategy that incorporates Bio-RoBERTa for textual feature extraction, a graph vision spatial channel attention transformer network for image feature learning, and cross-attention mechanisms for modality alignment. Further, the model employs principal component analysis (PCA) for dimensionality reduction and gradient boosting machines (GBMs) for semantic conflict resolution. Classification is performed using an ensemble of random forest KNN, proximal policy optimization (PPO), and a sparse radial basis function (RBF) kernel to ensure accuracy and interpretability. Experimental evaluation on publicly available datasets achieved 99.82% accuracy, a Dice coefficient of 98.7%, and significantly lower execution time compared to state-of-the-art methods. The results confirm the framework’s effectiveness in aligning and leveraging multi-modal data for precise classification of six GI diseases, offering a scalable and interpretable solution for enhanced clinical decision-making in gastroenterology.

## Introduction

The domain of gastrointestinal disease diagnosis has witnessed significant advancements with the integration of machine learning and deep learning techniques. Within this domain, Wireless Capsule Endoscopy (WCE) emerges as a critical sub-domain due to its non-invasive nature and ability to capture extensive visual data of the gastrointestinal tract^1^. Despite its potential, diagnosing diseases such as esophagitis, ulcerative colitis, ulcers, polyps, active bleeding, and vascular lesions poses substantial challenges^2^. Existing diagnostic frameworks struggle with issues like imprecise localization of abnormalities, variability in lesion appearance, and limited standardization in textual medical reports. The heterogeneity of data modalities, textual descriptions from medical reports and visual information from WCE images further exacerbates the problem^3^. These challenges hinder accurate diagnosis and necessitate the development of advanced multimodal systems capable of extracting, aligning, and leveraging meaningful features from diverse data sources to enhance diagnostic precision and efficiency^4^.

Feature extraction and fusion methods in current frameworks attempt to address the heterogeneity of text and image modalities but are constrained by significant limitations. In text feature extraction, conventional methods often rely on statistical models or shallow semantic techniques, which fail to capture the intricate and domain-specific nuances of medical language^5^. For instance, terminological variations in medical reports lead to inconsistent and ambiguous feature representations. Image-based extraction methods often struggle with WCE images, as subtle anomalies like faint lesions or small polyps are easily obscured by normal tissue, leading to missed diagnostic details^6^. Feature fusion techniques, while innovative, face the persistent challenge of aligning temporal and spatial inconsistencies between modalities. Textual descriptions, which often reference past medical events or treatments, are frequently out of sync with the real-time visual data captured by images^7^. Furthermore, overlapping information between text and images often leads to redundancy or conflict, complicating the fusion process. The inability to adequately harmonize these modalities or prioritize the most relevant features results in suboptimal feature representations, which compromise the effectiveness of downstream classification tasks, ultimately affecting diagnostic accuracy and reliability^8^.

Existing classification methods predominantly rely on single-modality models or basic ensemble techniques that inadequately address the complexities of multimodal data^9^. While models like Random Forests, Support Vector Machines (SVM), and standard Neural Networks have demonstrated utility, they often fail to effectively balance the influence of text and image modalities^10^. Challenges such as modality dominance, where one modality disproportionately affects classification decisions, and feature redundancy, where overlapping information from text and images leads to overfitting, remain unresolved^11^. These problems compromise classification models’ accuracy and resilience, which reduces their dependability for practical clinical applications^12^. Consequently, there is a pressing need for classification frameworks that can dynamically integrate and prioritize multimodal features while mitigating redundancy and conflict.

In recent years, several notable advancements in multimodal and multi-scale medical image processing have inspired progress in related domains. For instance, MLCA2F introduced a multi-level context attentional feature fusion mechanism for COVID-19 lesion segmentation from CT scans. These methods effectively integrate both global and local contextual cues to improve segmentation accuracy^13^. Similarly, BG-3DM2F leveraged bidirectional gated recurrent units with 3D multi-scale feature extraction to model spatial-temporal dependencies in Alzheimer’s disease diagnosis, illustrating the effectiveness of memory-based fusion in neurological applications^14^. In dermatology, a CAD system using a multi-layer feature fusion network improved the classification of dermoscopic images by capturing subtle differences between malignant and benign skin lesions^15^. In breast imaging, a region proposal-based multi-scale CNN effectively identified abnormalities by localizing key regions and handling lesion variability^16^. These approaches highlight the success of fusion and attention mechanisms in medical imaging, motivating their use in gastrointestinal diagnostics.

Transformer-based architectures, machine learning, and deep learning methods have shown promise in addressing the challenges of gastrointestinal disease diagnosis but face significant limitations. Transformers capture global dependencies and align text–image features well but are computationally intensive and prone to overfitting on small or imbalanced WCE datasets^17^. Machine learning techniques like gradient boosting and kernel-based methods are effective in handling specific feature interactions but struggle with scalability, feature redundancy, and heterogeneous data alignment across modalities^18^. Deep learning models like Vision Transformers (ViTs) and CNNs extract visual features effectively but require large labeled datasets and are sensitive to noise in real-world WCE images. These methods also face challenges in integrating multimodal data effectively, often overlooking contextual nuances or prioritizing one modality disproportionately^19^. These limitations highlight the need for hybrid frameworks that combine transformers, machine learning, and deep learning to enable more robust and accurate GI disease diagnosis.

### Challenges in multimodal gastrointestinal disease diagnosis

#### Challenges of feature extraction

WCE images poses significant challenges in feature extraction for diagnosing gastrointestinal diseases such as esophagitis, ulcerative colitis, ulcers, polyps, active bleeding, and vascular lesions. Textual data in medical reports often suffers from ambiguity and inconsistency due to non-standardized terminologies. For example, phrases like “redness in esophagus” or “inflammation of colon” and varying descriptors such as “erosive esophagitis” versus “GERD with erosion” hinder the precise extraction of textual features. This lack of standardization complicates the identification and correlation of relevant patterns across diverse medical records. On the other hand, visual data from WCE images presents challenges due to the overlap of features such as inflammation and redness. This makes distinguishing between conditions like esophagitis and ulcerative colitis particularly difficult. Additionally, the variability in lesion size, shape, and spatial distribution further complicates the extraction of distinctive features, especially in conditions such as ulcers and polyps. Small or flat polyps may resemble ulcers, creating ambiguity for standard feature extraction models. For active bleeding and vascular lesions, temporal misalignment between textual descriptions and real-time imaging adds another layer of complexity. Images capture the current state, while textual descriptions may refer to past events, creating a disconnection between modalities. Obstructed image features due to clots or pooled blood, coupled with faint vascular lesions often camouflaged by normal tissue, exacerbate the difficulty of accurate feature extraction.

#### Challenges of feature fusion

Integrating text and image data for gastrointestinal disease diagnosis is hindered by misalignment and inconsistencies between the two modalities. Temporal discrepancies arise when textual descriptions refer to past events, such as prior treatments or bleeding episodes, while images represent the current condition. Aligning these modalities to effectively correlate historical and real-time data is a non-trivial challenge. Spatial-context misalignment further complicates fusion, as images provide precise lesion location and depth, while text often lacks spatial details or includes generalized descriptions. Existing methods struggle to map spatial and temporal information between these two data types, which is critical for accurate fusion. Latent semantic conflicts also arise when text and image data present contradictory information. For instance, a medical report may suggest improvement, while the image reveals worsening pathology. Resolving such conflicts and identifying the most diagnostically relevant features require advanced prioritization techniques. Additionally, feature redundancy occurs when both modalities capture overlapping information, such as text describing ulcer size while the image visually represents it. Proper handling of redundancy is necessary to avoid overfitting and ensure that the fusion model emphasizes complementary, non-redundant features for accurate diagnosis.

#### Challenges of classification

Post-fusion classification is fraught with challenges related to modality imbalance and feature redundancy. Textual features offer valuable historical insights, such as prior treatments and symptoms, while image features provide detailed visual representations of the current condition, such as lesion presence and severity. However, these modalities often exert inconsistent influence on the classification decision. For example, a textual description of past treatment might outweigh critical visual evidence of new lesions, leading to misclassification. Balancing the influence of both modalities to ensure diagnostic relevance remains a key challenge. Redundant features further complicate classification. Overlapping information between text and image data, such as text describing ulcer size and corresponding image features, can lead to overfitting, where the model focuses excessively on repetitive patterns rather than complementary insights. To improve the robustness of the model and avoid biassed learning, redundant features must be properly identified and removed. Addressing these challenges is crucial for ensuring accurate and reliable classification, where both text and image modalities are appropriately prioritized and leveraged for optimal diagnostic outcomes.

### Major contributions of this research

- Multimodal feature extraction: A novel feature extraction pipeline is designed, combining Bio-RoBERTa for domain-specific text embedding and a Graph Vision Spatial Channel Attention Transformer Network for image processing, enabling accurate capture of semantic and spatial features relevant to GI disease diagnosis.
- Unified sparse transformer-based fusion network: The proposed recurrent multimodal principal gradient K-proximal sparse (RMP-GKPS) transformer fuses textual and visual features using sparse attention, cross-modal alignment, and recurrent temporal modeling, while incorporating PCA and GBM for dimensionality reduction and conflict resolution, achieving robust and efficient multimodal representation.
- Hybrid classification strategy with adaptive learning: A novel classification module integrates Random Forest KNN, Sparse RBF kernel, and proximal policy optimization (PPO) to dynamically balance modalities, reduce redundancy, and improve interpretability, resulting in enhanced diagnostic performance across six GI disease classes.

Overall, the novelty of the proposed framework lies in its system-level integration of sparse attention, recurrent modeling, and reinforcement learning, which collectively address challenges not sufficiently tackled by existing GI diagnostic models. The Sparse Transformer enables efficient attention computation, focusing only on diagnostically salient visual tokens and essential linguistic components in the clinical notes. The integration of Bio-RoBERTa, pre-trained on vast biomedical corpora, ensures contextually rich, domain-specific text embeddings, capable of capturing subtle semantic distinctions such as “erosive esophagitis” vs. “GERD with erosion.” The cross-attentional fusion module facilitates spatial and semantic alignment between modalities, while the inclusion of recurrence enables temporal tracking of disease progression across observations. Finally, the use of Proximal Policy Optimization (PPO) as a classification reinforcement module introduces an adaptive decision-making policy that dynamically adjusts based on modality reliability and noise patterns. This combined architecture has not been previously explored in GI disease diagnosis, making it novel both in design and diagnostic strategy.

This manuscript’s remaining contents are organised as follows: A brief review of earlier research is given in Sect. 2, the operation of the suggested technique is covered in Sect. 3, the results of the implementation and performance analysis of the suggested approach are shown in Sect. 4, and the study is ultimately concluded in Sect. 5.

## Literature survey

Trans MSF, a transformer and convolutional neural network-based CAD system, was proposed by Xuejiao Pang et al.^20^ to help endoscopists diagnose a variety of GI disorders. To record both global and local lesion information, our system built two feature extraction paths using different encoding techniques. To further enhance feature representations and lower memory and processing demands, downsampling was also incorporated into the transformer to extract global information at various scales. In order to reduce the loss of important information during spatial dimension changes and maintain target focus, a channel and spatial attention module with fewer parameters was created. The gathered feature data was then fed into a linear classifier for disease diagnosis after being fused via a feature fusion module. However, the downsampling approach in the transformer might inadvertently discard subtle and fine-grained lesion features, potentially leading to missed diagnoses in cases involving small or complex lesions.

To facilitate improvements in computer-assisted diagnosis and automatic detection of gastrointestinal disorders, Ayşe et al.^21^ proposed a novel architecture known as Spatial Attention ConvMixer (SAC). By adding a spatial attention mechanism (SAM), which gave each spatial location in the feature maps a certain amount of importance, this architecture improved upon the patch extraction method utilised in the ConvMixer architecture and enabled the network to selectively focus on the most informative regions. The SAC architecture demonstrated improved classification performance compared to several existing models, showcasing its ability to better capture disease-specific features. However, the heavy reliance on spatial attention mechanisms can make the model more vulnerable to inaccuracies when dealing with images containing inconsistent or noisy spatial features, potentially leading to misclassifications in challenging scenarios.

Salman et al.^22^ presented a method that uses a vision transformer and transfer learning model to identify gastrointestinal tract illnesses and aid in medical diagnosis based on attributes taken from endoscopic pictures. Vision transformers were employed due to their demonstrated success in challenging image classification tasks, and the approach achieved high accuracy in detecting gastrointestinal diseases. This method utilized the ability of vision transformers to analyze the entire image context, making it particularly effective for feature extraction and categorization. However, the substantial computational overhead associated with vision transformers can hinder their real-time deployment and make them less accessible for use in low-resource environments or portable diagnostic devices.

In order to concurrently focus on local features and global attention, Shibin et al.^23^ introduced FLATer, a transformer-based model that is quick, light, and accurate. It combines a spatial attention block, a visual transformer module, and a residual block. This design made use of the advantages of vision transformers and convolutional neural networks. Two subtasks were used to classify endoscopic images: multi-class classification to detect certain diseases such ulcerative colitis, polyps, and oesophagitis, and binary classification to distinguish between normal and diseased images. FLATer demonstrated a better accuracy in binary classification and multi-class classification. However, the reliance on task decomposition into separate classifications would lead to limitations in handling complex cases where diseases overlap or coexist, potentially reducing its applicability in real-world clinical diagnostics.

A new transformer-based deep neural network that can execute several tasks at once was introduced by Pham et al.^24^, allowing for the precise detection of colon polyps and lesions in the upper gastrointestinal tract. This method improved the network’s representation capability by utilising the MiT backbone and a feature alignment block in conjunction with a novel global context-aware module. The architecture achieved significant improvements in performance across various endoscopic diagnostic tasks, showcasing its adaptability and robust feature learning. Extensive experimental results highlighted its superior effectiveness compared to other state-of-the-art methods. However, the intricate design of the architecture results in increased training and inference times, posing challenges for integration into real-time diagnostic workflows or environments with limited computational resources.

In order to diagnose ulcerative colitis, polyps, and dyed-lifted polyps using wireless capsule endoscopy images, Hassaan et al.^25^ proposed four multi-classification deep learning (DL) models: Vgg-19 + CNN, ResNet152V2, Gated Recurrent Unit (GRU) + ResNet152V2, and ResNet152V2 + Bidirectional GRU (Bi-GRU). These models were applied to a variety of publicly accessible databases. These models demonstrated significant potential in addressing classification challenges in gastrointestinal disease diagnosis. However, the approach was limited by the lack of robust multi-modal feature integration, which could potentially enhance the accuracy and reliability of the diagnostic predictions.

GastroNet, an attention-based system, was proposed by Noor et al.^26^ for the classification of gastrointestinal illnesses. To improve intra-class dissimilarity, sick spots within images were highlighted using an attention technique. Superimposed images were created by fusing attention-enhanced images with the original input images. Data augmentation techniques were used to boost the training of a fine-tuned model. From these overlay images, deep CNN-based features were retrieved, and a feature selection technique based on cosine similarity was used to minimise the dimensionality of the extracted features. Lastly, several machine learning classifiers were used to categorise diseases. However, the proposed method was limited by its reliance on manual tuning for feature selection and classifier optimization, which could affect scalability and adaptability to different datasets.

Prior to the Denoising Convolutional Neural Networks (DnCNNs) approach, which was suggested as a pre-processing tool for endoscopic image classification to help with gastrointestinal disease identification, Ahmed^27^ created AlexNet with no data augmentation and fine-tuned settings. Eight classes were identified from the Kvasir dataset for endoscopic images using a combination of classification techniques and the denoising pre-processing step. However, the approach was limited by the absence of data augmentation, which could potentially hinder its ability to generalize effectively to diverse and complex real-world datasets.

Ozbay et al.^28^ used a long-range transformer model in conjunction with a method for segmenting GI tract regions in WCE image scans. The transformer block was subjected to a split token embedding technique in order to generate local information with surrounding pixels and patches in the design. Along with the Residual block and Inception module, feature learning was carried out utilising a cross-channel approach to improve model training efficacy and capture complex anatomical aspects of the GI tract. The WCE Curated Colon Disease (WCECCD) dataset, which consists of four image classes covering three GI tract disorders and normal circumstances, was used to test the suggested architecture. However, the approach was constrained by its reliance on the WCECCD dataset, which might not accurately reflect the variety of GI tract conditions seen in more general clinical settings.

Ghany et al.^29^ developed the Intelligent Learning Rate Controller (ILRC) mechanism, which adaptively modifies the learning rate (LR) in response to training progress, to maximise the training of DL models. This method was created to reduce the chance of overfitting and increase convergence speed. Four DL models—EfficientNet-B0, ResNet101v2, InceptionV3, and InceptionResNetV2—were subjected to the ILRC. Techniques like transfer learning, layer freezing, fine-tuning, residual learning, and contemporary regularisation methods were used to further enhance these models. Two datasets, Kvasir-Capsule and KVASIR v2, which contained images from wireless capsule endoscopy, were used to evaluate the models. According to the results, the models that used ILRC performed noticeably better in terms of accuracy than the state-of-the-art techniques. However, a limitation of the ILRC mechanism is its dependency on the specific architecture and dataset. Performance may vary across different configurations and applications, potentially limiting its generalizability.

While prior models in gastrointestinal disease classification have achieved promising results, they predominantly rely on single-modality inputs or simplistic fusion strategies that fail to address deeper cross-modal conflicts and redundancies. Many lack mechanisms to resolve inconsistencies between textual and visual data or dynamically adjust feature weighting based on context. Additionally, conventional architectures often struggle with aligning temporal cues from medical history with spatial features from endoscopic images. The proposed work addresses these limitations by introducing a cross-attentional, sparse, and recurrent multimodal fusion framework. This framework not only aligns heterogeneous data effectively but also incorporates conflict resolution and dynamic feature prioritization to enhance diagnostic accuracy and robustness.

### Proposed methodology

**Fig. 1:**
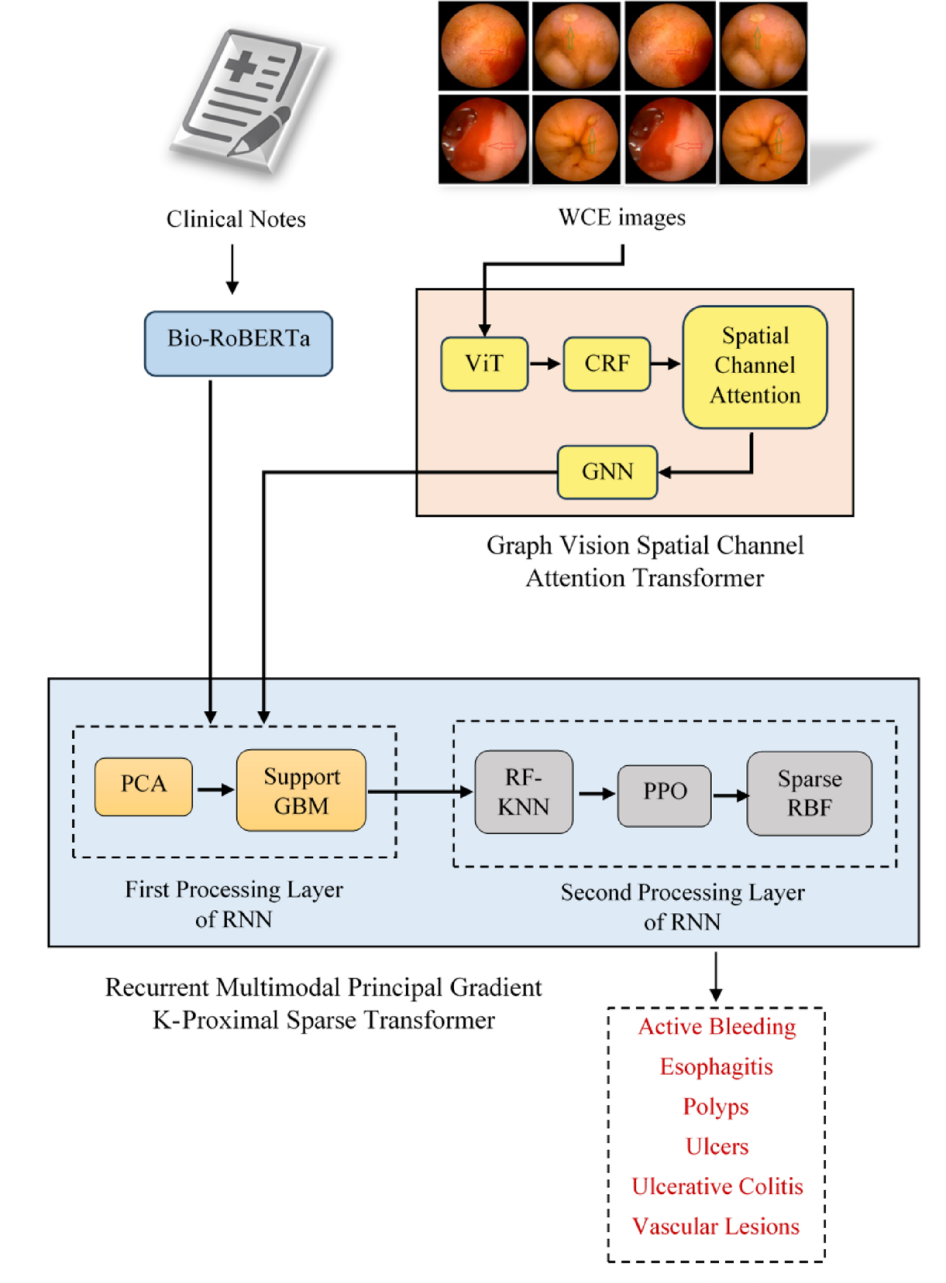
Overall architecture of the proposed RMP-GKPS-transformer approach.

The proposed research introduces an Efficient Transformer-Based Multi-Modal Fusion Framework designed to address the intricate challenges of gastrointestinal disease diagnosis from WCE data. The approach leverages advanced transformer architectures, graph-based mechanisms, and reinforcement learning to effectively extract, fuse, and classify multi-modal data, encompassing both textual and visual modalities. The key components of the framework are systematically developed to ensure precise, robust, and interpretable diagnostic outcomes. The overall architecture of the proposed methodology is shown in (Fig. 1).

Feature extraction from text and image data is the foundation of the proposed approach. Textual data from medical reports often suffers from contextual ambiguity and terminological inconsistencies. To address this, a Bio-RoBERTa model is utilized to generate high-dimensional embeddings that capture both semantic and syntactic nuances. This ensures the accurate representation of complex medical terminology and relationships, such as distinguishing minute diagnostic differences between terms like “erosive esophagitis” and “GERD with erosion”. For visual image data, the proposed framework incorporates a Graph Vision Spatial Channel Attention Transformer Network. This integrates Vision Transformers (ViT) for global and local feature representation, Conditional Random Fields (CRF) for spatial coherence, and a Spatial Channel Attention mechanism to emphasize critical regions. This ensures precise identification of indirect and complex features, such as faint vascular lesions or small polyps, even under challenging imaging conditions like clots or pooled blood. The integration of Graph Neural Networks (GNNs) further refines these features, representing spatial relationships in a graph-like format for alignment with textual insights.

The extracted features are then fused using a novel RMP-GKPS-Transformer Network, which effectively integrates the strengths of text and image data while addressing cross-modal challenges. A Multimodal Transformer resolves temporal and spatial misalignments by employing cross-attention mechanisms. This aligns past textual descriptions (e.g., prior treatment or symptoms) with real-time visual data. To tackle dimensionality and redundancy, Principal Component Analysis (PCA) filters out irrelevant or overlapping features, ensuring the selection of the most salient characteristics. Latent semantic conflicts, such as contradictory information between modalities, are resolved through Support Gradient Boosting Machines (GBM). This prioritizes the most consistent and diagnostically relevant features. The fused features are then processed through a Recurrent Neural Network (RNN), which captures temporal dynamics critical for progressive conditions like active bleeding or ulceration. This multi-layered fusion strategy ensures robust alignment and integration of modalities, paving the way for accurate diagnostic outcomes.

Finally, to address the challenges of misclassification and modality imbalance, the fused features are classified using an ensemble approach within the RNN’s second hidden layer. The classification module integrates random forest KNN, proximal policy optimization (PPO), and a sparse radial basis function (RBF) kernel. Random Forest KNN ensures a balanced influence of textual and visual features, leveraging decision trees for feature importance evaluation and KNN for local consistency. Proximal policy optimization (PPO) dynamically adjusts the prioritization of features during classification, ensuring that decisions reflect the most current and relevant diagnostic evidence. The Sparse RBF kernel eliminates redundant or overlapping features by focusing on unique and complementary information, preventing overfitting and enhancing generalization. Together, these components enable the classification module to handle modality conflicts, resolve feature redundancy, and achieve accurate and interpretable predictions.

This proposed approach ensures that the challenges associated with feature extraction, fusion, and classification are systematically addressed, enabling comprehensive and accurate gastrointestinal disease diagnosis. The novel combination of transformer-based architectures, graph-based processing, and reinforcement learning creates a robust framework capable of handling the complexities of multi-modal medical data.

### Feature extraction

Feature extraction forms the base of the proposed approach, leveraging domain-specific techniques to extract rich, representative features from both textual and visual data. This stage operates in two distinct but interconnected phases: textual feature extraction and visual feature extraction, ensuring comprehensive representation across modalities. These phases are further explained in the upcoming sections.

#### Textual feature extraction

Non-standardized terminologies cause ambiguity and inconsistencies in textual data from medical reports. To address this, the Bio-RoBERTa model, a transformer-based architecture pre-trained on biomedical text, is employed. The input text $\:T=\left\{{t}_{1},\:{t}_{2},\:\dots\:,{t}_{n}\:\right\}$, where $\:{t}_{i}$ represents individual tokens, is fed into Bio-RoBERTa, which outputs high-dimensional embeddings, $\:{H}_{t}=\left\{{h}_{1},\:{h}_{2},\:\dots\:,{h}_{n}\:\right\}$, where, $\:{h}_{i}\in\:{R}^{d}$. Here, $\:{H}_{t}$ represents the contextual embeddings capturing semantic and syntactic nuances, ensuring accurate differentiation of terms like “erosive esophagitis” and “GERD with erosion”. $\:{R}^{d}$ refers to the dimensionality of the high-dimensional embeddings generated by the Bio-RoBERTa model for each token in the input text. These embeddings are aggregated using mean-pooling which is mathematically expressed as given by Eq. (1),

1 $$\:{E}_{T}=\frac{1}{n}\sum\:_{i=1}^{n}{h}_{i,\:\:\:\:\:\:\:\:\:\:\:\:\:\:\:\:\:\:\:\:\:\:}{E}_{T}\in\:{R}^{d}$$

Where, $\:{E}_{T}$ serves as the global textual representation for downstream alignment with visual data.

#### Visual feature extraction

For image data, the Graph Vision Spatial Channel Attention Transformer Network is introduced to address the complexities of Wireless Capsule Endoscopy images. In this phase, initially, the WCE images $\:{I}_{P}\in\:{R}^{H\times\:W\times\:C}$ are divided into non-overlapping patches of size $\:P\times\:P$, resulting in $\:N=\frac{H.W}{{P}^{2}}$ patches. Each patch is linearly embedded into a token represented as $\:\:{X}_{P}=Linear\:\left({I}_{P}\right),\:\:where$ $\:{X}_{P}\in\:{R}^{d}$. These tokens are then passed through the Vision Transformer resulting in visual embedding which is mathematically expressed as shown in Eq. (2)

2 $$\:{E}_{I}=ViT\:\left(Linear\:\left({I}_{P}\right)\right)$$

These embeddings generated by the ViT captures both global and local visual features. To ensure spatial coherence, CRFs are applied on top of the ViT outputs. CRFs refine the segmentation by modeling spatial relationships between neighbouring regions, ensuring accurate boundary delineation of lesions which is mathematically represented as given in Eq. (3)

3 $$\:P\left(y|z\right)=\frac{1}{Z\left(x\right)}exp\left(\sum\:_{i}{\varPsi\:}_{i}({x}_{i},{y}_{i})+\sum\:_{i,j}{\varPsi\:}_{ij}({y}_{i},{y}_{j})\right)$$

where $\:{\varPsi\:}_{i}$ is the unary potential to capture individual patch characteristics, $\:{\varPsi\:}_{ij}$​ is the pairwise potential encoding spatial relationships, and $\:Z\left(x\right)$ is the partition function. Then, a spatial channel attention mechanism is incorporated to dynamically weight regions and color channels, emphasizing fine features like faint bleeding or small polyps. At this stage, an enhanced feature map is obtained which is mathematically represented as provided in Eq. (4),

4 $$\:{H}_{A}=A\odot\:{E}_{I}$$

where, $\:A$ is the attention weight that highlights the most diagnostically relevant regions and $\:\odot\:$ denotes the element-wise multiplication. This enhanced feature map is then transformed into a graph structure $\:G=(V,\:E)$ using GNN where, $\:V$ represents image patches, and $\:E$ denotes spatial relationships. GNNs refine the features using graph convolutions as represented by Eq. (5),

5 $$\:{H}_{G}^{l+1}=\sigma\:\left({D}^{-1}{A}_{d}.{H}_{G}^{l}.{W}^{l}\right),\:\:\:\:\:\:\:\:l\ge\:0$$

where, $\:{A}_{d}$is the adjacency matrix, $\:D$ is the degree matrix, $\:{W}^{l}$ is the learnable weight matrix, $\:\sigma\:$ and is the activation function. The output $\:{H}_{G}$ is a graph-structured representation of visual features. The final textual feature vector $\:{E}_{T}$ and the visual feature matrix $\:{H}_{G}$ are outputted as the extracted features. These are aligned and fused in subsequent stages to enable accurate gastrointestinal disease diagnosis.

### Multi-modal feature fusion

The multi-modal feature fusion stage is designed to integrate textual and visual features extracted from the previous stage, ensuring their alignment and mitigating cross-modal challenges such as spatial, temporal, and semantic inconsistencies. This stage uses a Recurrent Multimodal Principal Gradient K-Proximal Sparse Transformer Network, incorporating cross-attention mechanisms, dimensionality reduction, and conflict resolution strategies to achieve robust integration of modalities.

Although Gradient Boosting Machines (GBMs) are inherently robust to feature redundancy, the integration of Principal Component Analysis (PCA) prior to GBM provides multiple benefits in the context of high-dimensional multi-modal fusion. PCA performs an orthogonal linear transformation of the fused feature space, preserving maximum variance along principal components while discarding noise and collinearity. This reduces input dimensionality to GBM, enabling faster convergence, lower memory usage, and better feature decorrelation for more effective decision tree construction. PCA ensures that only the most diagnostically relevant features are retained, which complements GBM’s iterative refinement mechanism, ultimately improving classification generalization.

The goal of this fusion stage is to fuse $\:{E}_{T}$ and $\:{H}_{G}$ into a single multi-modal feature representation that retains the strengths of both modalities while resolving inconsistencies. The Cross-Attention operation is defined as given by Eqs. (6),

6 $$\:{A}_{c}=softmax\:\left(\frac{Q{K}^{T}}{\sqrt{{d}_{k}}}\right),\:\:\:\:\:\:\:\:\:{A}_{c}\in\:{R}^{1\times\:N}$$

where, $\:Q={W}_{q}{E}_{T}$ and $\:K={W}_{k}{H}_{G}$ are the query and key matrices respectively with learnable weight matrices and $\:{d}_{k}$ is the dimensionality of the key. The cross-attended feature is computed using Eqs. (7),

7 $$\:{F}_{CA}={A}_{c}.\:V={A}_{c}.{W}_{v}{H}_{G}$$

where, $\:{W}_{v}$ is the learnable weight of the value matrix $\:V$. The resulting ​$\:{F}_{CA}$ aligns textual insights with visual regions, resolving spatial misalignments and reinforcing mutual context.

To integrate temporal dependencies, for example, changes in ulceration progression, recurrence is introduced post fusion through an RNN layer that operates on the sequence of cross-attended fused tokens $\:{F}_{CA}$. After aligning visual and textual features using cross-attention, the sequence of fused embedding is passed to the RNN to capture temporal dependencies and progression dynamics across observations. For instance, in longitudinal studies where multiple WCE frames or repeated notes are available for the same patient, the RNN models how conditions evolve (e.g., increasing inflammation, new bleeding). This enhances the model’s understanding beyond static classification, allowing it to support progression-aware predictions. The hidden state updates of the RNN encode prior diagnostic context, which is especially useful for diseases like ulcerative colitis or vascular lesions where the appearance can change over time. This is represented by Eq. (8),

8 $$\:{H}_{RNN}^{t}=\sigma\:\:\left({W}_{h}{H}_{RNN}^{t-1}+{W}_{x}{F}_{CA}^{t}+b\right),\:\:\:\:\:\:\:\:\:\:\:\:\:\:t\in\:[1,\:T]$$

where, $\:T$ is the sequence length, $\:{H}_{RNN}^{t}$ is the hidden state at the time $\:t$, $\:\sigma\:$ is the activation function, and $\:{W}_{h}$ and $\:{W}_{x}$ are the learnable weight parameters. To handle the high-dimensional nature of $\:{F}_{CA}$ and $\:{H}_{RNN}$​, a Principal Component Analysis (PCA) is employed to filter out redundant and irrelevant features. PCA projects the feature space onto a lower-dimensional subspace while preserving the most significant variance. Given a feature matrix $\:{F}_{CA}\in\:{R}^{N\times\:d}$, the covariance matrix is computed as, $\:\epsilon\:=\frac{1}{N}{{F}_{CA}}^{T}{F}_{CA}$. Eigen decomposition of this covariance matrix $\:\epsilon\:$ yields eigenvalues $\:{\lambda\:}_{1},\:{\lambda\:}_{2},\dots\:,{\lambda\:}_{d}$​ and eigenvectors $\:{u}_{1},\:{u}_{2},\dots\:,{u}_{d}$​. The reduced feature representation is given by $\:{F}_{PCA}={F}_{CA}.{U}_{k}$, where, $\:{U}_{k}={u}_{1},\:{u}_{2},\dots\:,{u}_{d}$ and $\:k\le\:d$. Here, $\:k$ is selected based on the explained variance threshold.

To address latent semantic conflicts i.e., contradictory or misaligned information between modalities, Support Gradient Boosting Machines (GBM) are employed. GBMs iteratively refine the fused representation by prioritizing diagnostically consistent features. Given an input feature ​$\:{F}_{PCA}$ and a target diagnostic label $\:y$, GBM minimizes the loss function $\:L$ by adding weak learners $\:{h}_{m}$​ sequentially and the final fused representation is given by Eqs (9),

9 $$\:{F}_{Final}={F}_{GBM}^{\left(m\right)}={F}_{GBM}^{(m-1)}+\alpha\:{h}_{m}$$

where, $\:\alpha\:$ is the learning rate, and $\:m$ refers to the index of the current iteration or stage in the GBM process. This final fused multi-modal feature vector representation integrates textual and visual insights, resolving misalignments and conflicts, and is optimized for classification in the subsequent stage.

Compared to traditional full-attention Transformers that compute pairwise attention across all tokens with $O~\left( {n^{2} } \right)$ complexity, the Sparse Transformer reduces this complexity to $\:O\:\left(n\:log\:n\right)$ by restricting attention to a subset of positions. In the context of medical image and text fusion, this sparsity is particularly advantageous for high-resolution wireless capsule endoscopy (WCE) images and long clinical text sequences. While standard Transformers may become computationally intensive with such data, the Sparse Transformer maintains model scalability without sacrificing the contextual richness required for diagnosis.

For visual features, sparse attention allows the model to focus on diagnostically significant image patches (e.g., ulcer regions, vascular lesions) while ignoring redundant or background regions. In textual inputs, it emphasizes disease-specific phrases (e.g., “mucosal erosion” or “bleeding site”), bypassing less relevant tokens. This selective processing reduces overfitting, improves memory efficiency, and accelerates training crucial for real-time medical applications. The fusion of sparsely attended features leads to a more compact and meaningful multi-modal representation, significantly enhancing both diagnostic interpretability and performance.

The proposed Multi-Modal Feature Fusion algorithm is shown in (Algorithm 1).

**Algorithm 1:**
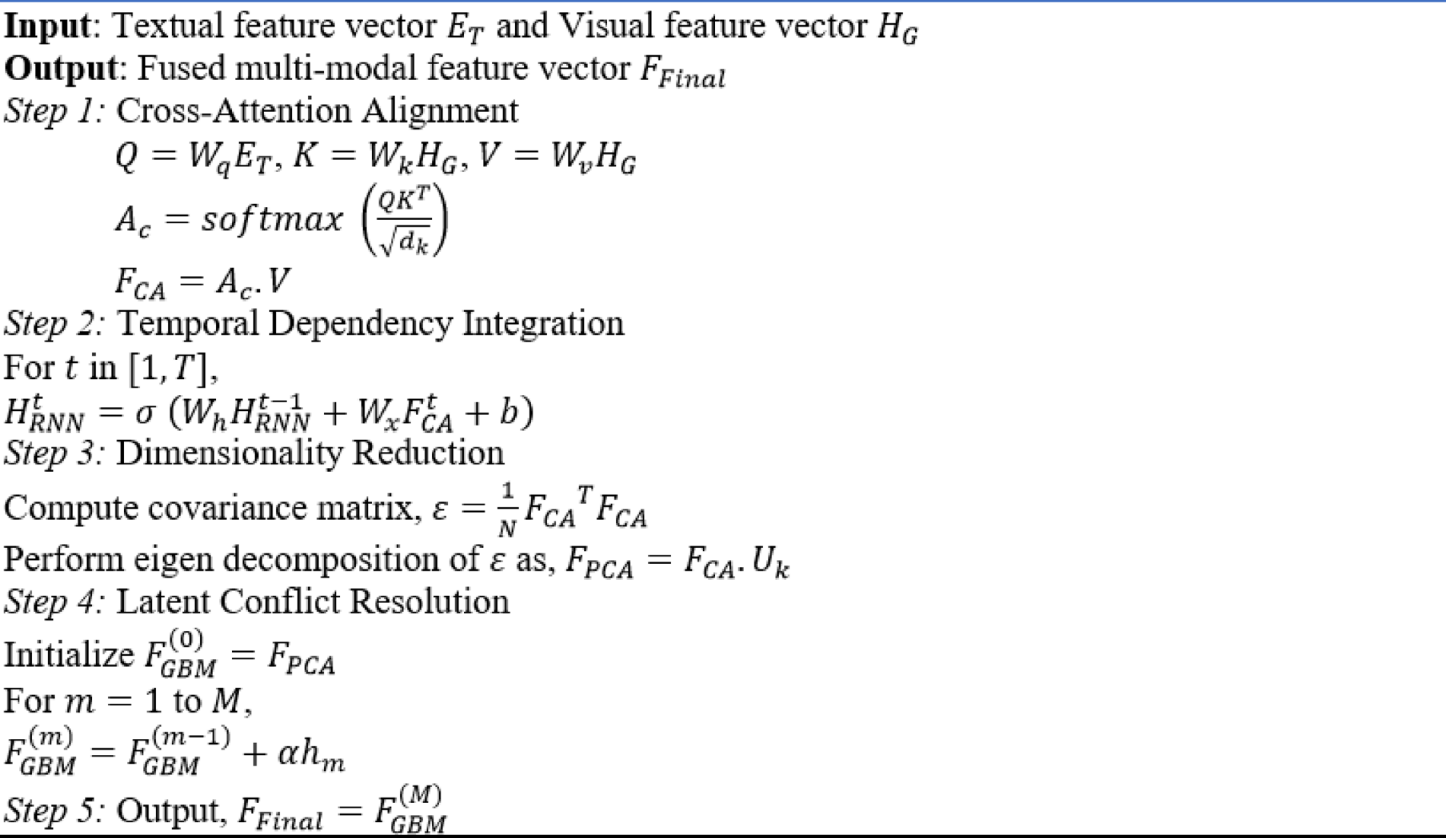
Multi-modal feature fusion.

By employing these advanced techniques, the fusion stage ensures robust alignment and integration, enabling the model to leverage the complementary strengths of text and image data for accurate diagnostic predictions.

### Classification

The classification stage is the final step of the proposed framework, leveraging ensemble learning techniques and reinforcement-based decision models to address challenges like modality imbalance, misclassification, and feature redundancy. This stage takes the fused multi-modal feature vector $\:{F}_{Final}$ from the previous stage as input and delivers diagnostic predictions for specific gastrointestinal diseases, including esophagitis, ulcerative colitis, ulcers, polyps, active bleeding, and vascular lesions. By integrating Random Forest KNN, Proximal Policy Optimization (PPO), and a Sparse Radial Basis Function (RBF) kernel, this stage ensures accurate and interpretable classification results.

Random Forest KNN is employed to balance the influence of textual and visual modalities. Random Forest evaluates feature importance by constructing an ensemble of decision trees, ensuring that the most diagnostically relevant features are prioritized. Meanwhile, the K-Nearest Neighbors algorithm ensures local consistency, particularly beneficial in cases where subtle visual features like faint vascular lesions or small polyps require context-aware classification.

Proximal Policy Optimization is employed to introduce policy-based learning in the classification stage, allowing the model to adaptively prioritize features from different modalities. Unlike traditional classifiers such as MLP or softmax, which apply static weights and lack adaptability, PPO treats classification as a sequential decision-making process. It optimizes a surrogate loss function that prevents over-adjustment of the policy (via clipping), ensuring stable convergence even under noisy or ambiguous input conditions. For example, in scenarios where the textual description suggests remission, but the image shows active bleeding, PPO learns to reweight visual features based on the current policy gradient, thereby avoiding misclassification. This adaptive behavior is governed by reward signals based on diagnostic consistency, enabling real-time policy adjustment. Such dynamic re-calibration is essential in medical diagnostics, where conflicting evidence across modalities is common.

To prevent overfitting and improve generalization, a Sparse RBF kernel is incorporated into the classification module. This kernel emphasizes unique and complementary features while eliminating redundant information. For instance, only the most distinctive features are used in the diagnosis of ulcers and polyps, when visual features and textual descriptions may overlap, according to the Sparse RBF kernel. The refined feature vector $\:{F}_{SparseRBF}$ is fed into a softmax classifier to produce diagnostic predictions as represented by Eq. (10) for gastrointestinal diseases$\:{\{y}_{1},\:{y}_{2},\dots\:,{y}_{c}\}$.

10 $$\:P\left({y}_{c}|{F}_{SparseRBF}\right)=\frac{\text{exp}\left({{w}_{c}}^{T}{F}_{SparseRBF}+{b}_{c}\right)}{{\sum\:}_{c=1}^{C}\text{exp}\left({{w}_{c}}^{T}{F}_{SparseRBF}+{b}_{c}\right)}$$

where $\:C$ is the total number of disease classes (esophagitis, ulcerative colitis, ulcers, polyps, active bleeding, and vascular lesions), $\:{w}_{c}$​ and ​$\:{b}_{c}$ are learnable parameters for class $\:c$. The algorithm for this ensemble Learning and reinforcement-based classification approach is shown in (Algorithm 2).

**Algorithm 2:**
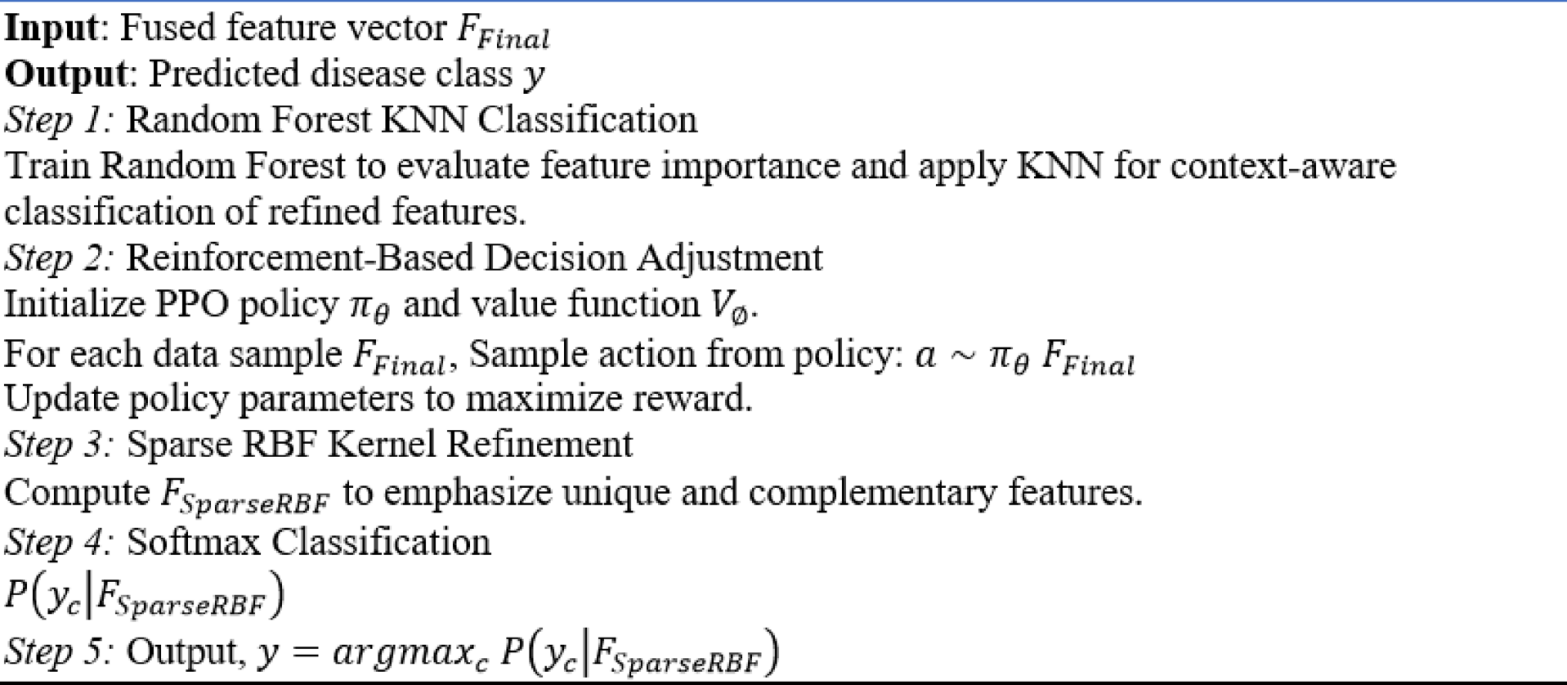
Classification with ensemble learning and reinforcement-based decision models.

The proposed classification framework effectively addresses disease-specific challenges by leveraging modality-specific strengths and advanced feature prioritization techniques. For esophagitis and ulcerative colitis, it ensures accurate discrimination of subtle inflammatory patterns, such as redness and inflammation, through the integration of complementary textual and visual data. In the case of ulcers and polyps, the framework overcomes size and shape variability as well as overlapping features by employing feature prioritization and redundancy mitigation mechanisms. For active bleeding and vascular lesions, it resolves text-image temporal conflicts and highlights faint features, enabling accurate detection despite obstructions like clots or pooled blood.

## Results and discussion

This section provides a comprehensive overview of the dataset description, system specifications, and implementation details for the proposed approach. It includes the evaluation of performance metrics followed by a detailed comparison with state-of-the-art methods. The discussion highlights the significance of the results and the improvements achieved in gastrointestinal disease classification.

### Dataset description

The datasets used in this study include both text and image data, focusing on six gastrointestinal diseases: esophagitis, ulcerative colitis, ulcers, polyps, active bleeding, and vascular lesions. The text dataset was sourced from the publicly available Kaggle dataset, which contains abstracts from five different conditions, including digestive system diseases cardiovascular diseases, neoplasms, nervous system diseases, and general pathological conditions. From this dataset, the clinical notes and diagnostic observations for gastrointestinal tract diseases were separately extracted and pre-processed to address inconsistencies, remove irrelevant data, and standardize terminology for effective model training and evaluation. The processed text was then converted into numerical feature vectors using the proposed Bio-RoBERTa model, enabling robust semantic representation for fusion and classification.

(https://www.kaggle.com/datasets/chaitanyakck/medical-text)

The image dataset was obtained from the Kaggle-hosted Kvasir dataset.

(https://www.kaggle.com/datasets/meetnagadia/kvasir-dataset).

Preprocessing of these images involved resizing them to a uniform resolution of 224 × 224 pixels, normalizing pixel values for consistency, and applying data augmentation techniques such as rotation, flipping, and brightness adjustments to improve model generalization and robustness. Then, these pre-processed gastrointestinal endoscopy images containing a total of 8,000 were categorized into six disease-specific classes: 1,200 images for esophagitis, 1,000 for ulcerative colitis, 1,600 for ulcers, 1,800 for polyps, 1,200 for active bleeding, and 1,200 for vascular lesions. Of these, 6,400 images (80%) were used for training, and 1,600 images (20%) were allocated for testing, with 267 images per class for active bleeding, esophagitis, polyps, ulcers, and ulcerative colitis, and 265 images for vascular lesions. A statistical summary of the dataset used is shown in (Table 1).

**Table 1:** Dataset statistical summary table.

Disease class	Number of training images	Number of testing images	Total images
Esophagitis	933	267	1200
Ulcerative colitis	733	267	1000
Ulcers	1333	267	1600
Polyps	1533	267	1800
Active bleeding	933	267	1200
Vascular lesions	935	265	1200
Total	6400	1600	8000

Together, the text and image datasets provide complementary information, with textual data offering contextual insights and image data contributing rich visual details, facilitating a comprehensive evaluation of the proposed multi-modal framework for gastrointestinal disease diagnosis.

### Implementation and performance evaluation results

The experimental outcomes of the proposed multi-modal fusion based classification framework are discussed in this section. The experiments in this study were conducted on a 64-bit Windows 10 Operating System with 32 GB of RAM and a 1 TB hard drive. The coding for implementing the proposed approach was carried out using the Python programming language, ensuring compatibility with various libraries and frameworks necessary for multi-modal data processing, feature fusion, and classification tasks. This system setup provided sufficient computational resources to handle the extensive training of the model over 50 epochs. This allows for efficient processing of both text and image data while ensuring smooth execution of the proposed algorithms.

**Fig. 2:**
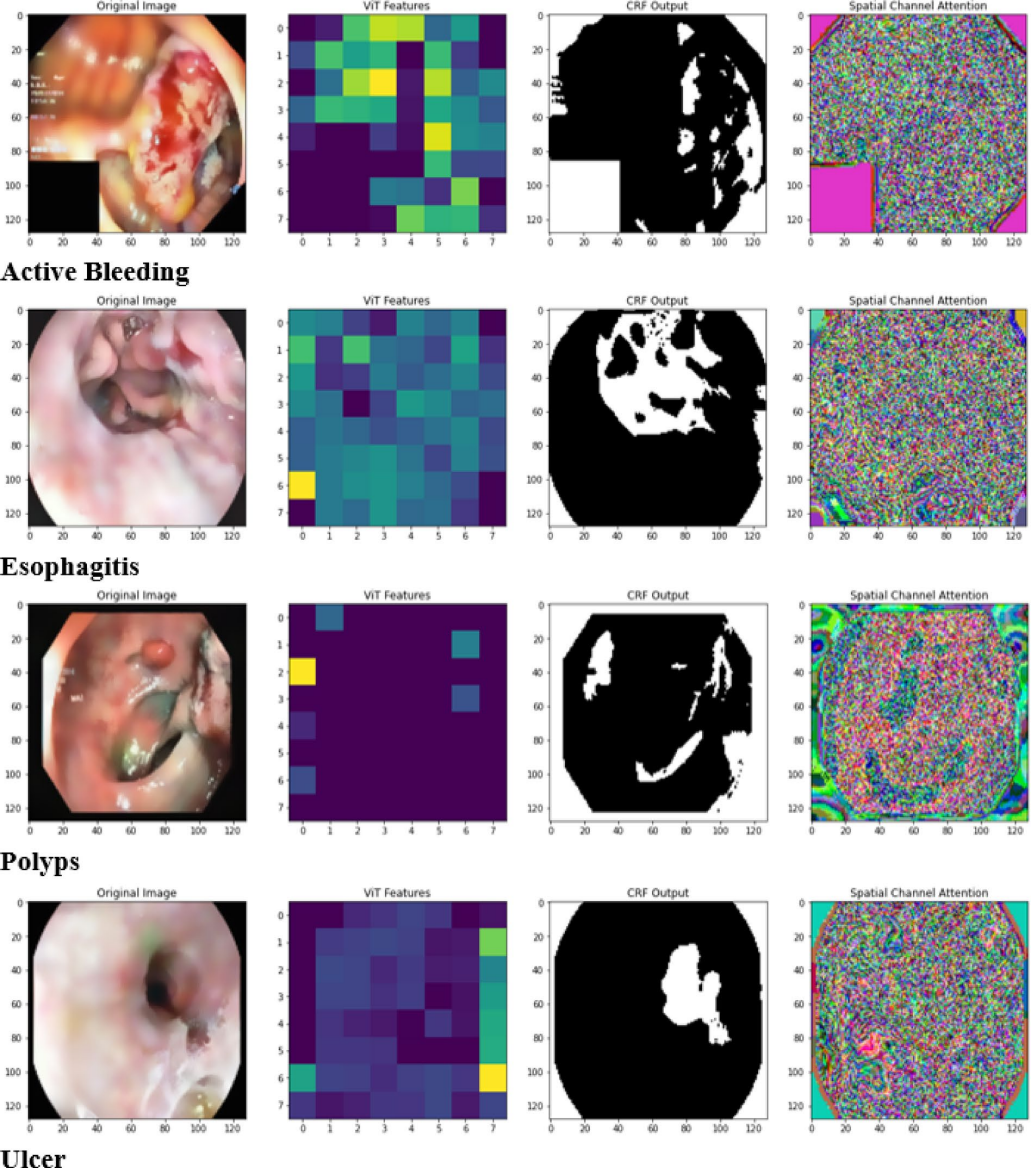

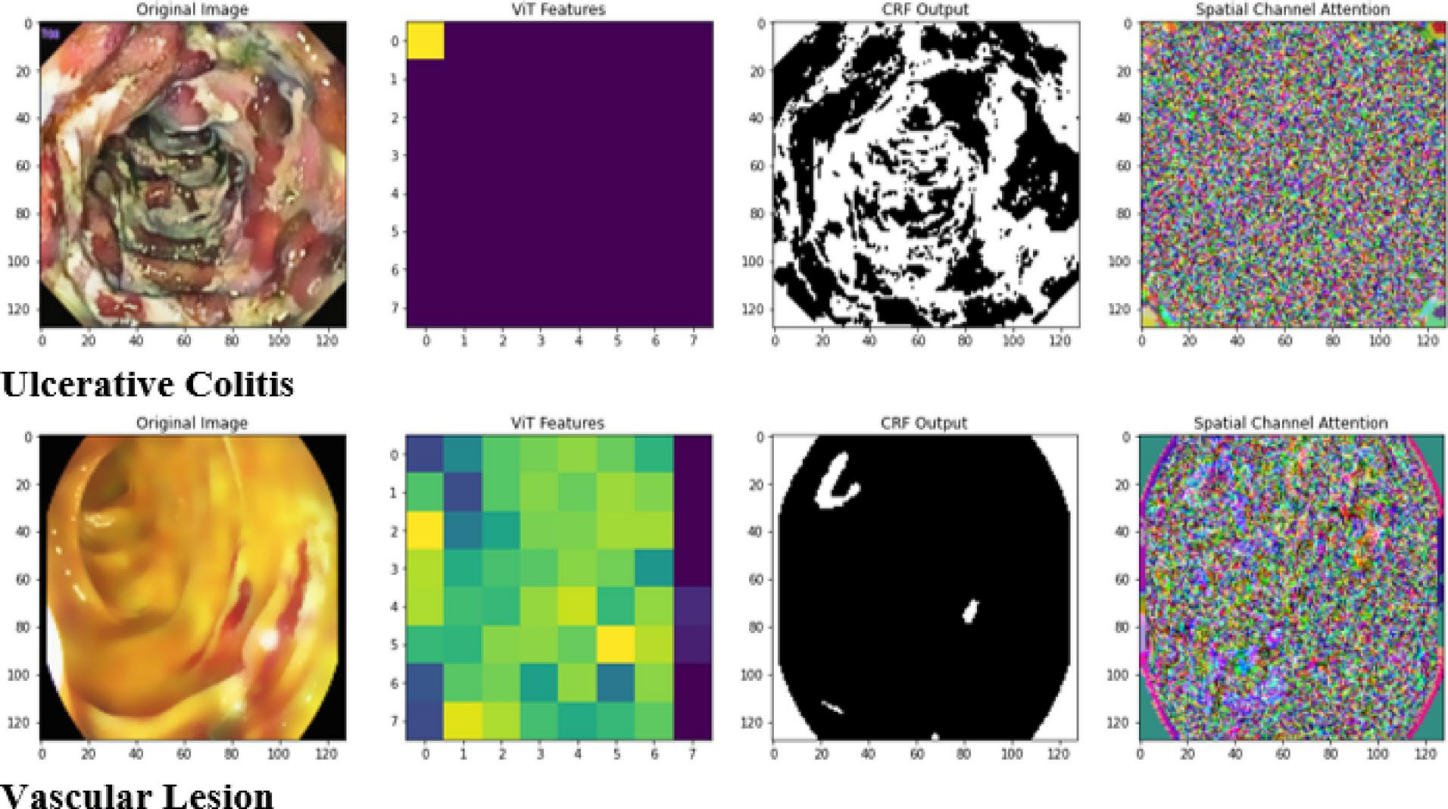
Implementation output of the proposed RMP-GKPS-transformer network.

The implemented output of the WCE image data for each gastrointestinal disease, active bleeding, esophagitis, polyps, ulcers, ulcerative colitis and vascular lesions is provided in (Fig. 2). The outputs include the original images, extracted ViT features for capturing global context, CRF outputs for refined spatial segmentation, and Spatial Channel Attention maps highlighting disease-relevant regions. This comprehensive visualization underscores the ability of the proposed RMP-GKPS-Transformer Network to integrate spatial, channel, and contextual features for precise and interpretable disease diagnosis.

**Fig. 3:**
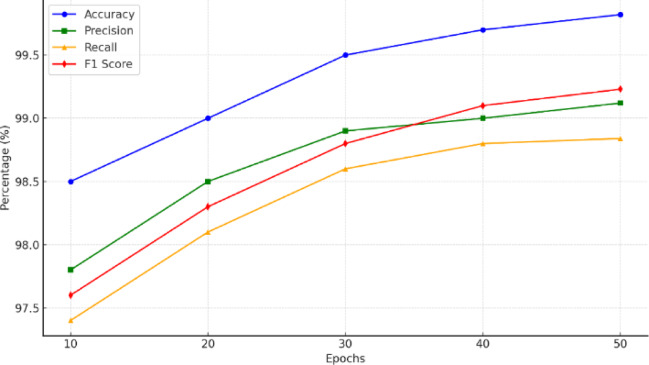
Performance metrics of the RMP-GKPS-transformer approach over epochs.

Figure 3 shows the performance results of the proposed approach in terms of accuracy, precision, recall and F1 score. The achieved accuracy of 99.82% highlights the exceptional performance of the proposed approach in correctly classifying gastrointestinal diseases across six conditions, demonstrating the overall effectiveness of the model. This integration effectively balanced the influence of textual and visual features, enabling the model to make informed decisions and minimize misclassifications. The precision of 99.12% from Fig. 3 indicates the model’s high reliability in identifying true positive cases, minimizing false positives with a Sparse Radial Basis Function (RBF) kernel, which prioritized discriminative features while mitigating redundancy. This ensured that the model focused on the most relevant characteristics of each disease, reducing false alarms and enhancing classification reliability.

Similarly, the recall of 98.84% underscores the model’s ability to identify the majority of actual disease cases, minimizing false negatives critical in medical diagnostics where undetected conditions can have severe consequences. The F1 Score of 99.23% highlights the balance between precision and recall, critical for avoiding both false positives and false negatives, thus ensuring robust and reliable performance. The combined use of Random Forest KNN, Sparse RBF kernel, and PPO allowed the model to handle trade-offs effectively, resulting in a diagnostic framework that is both accurate and dependable for real-world medical applications.

**Fig. 4:**
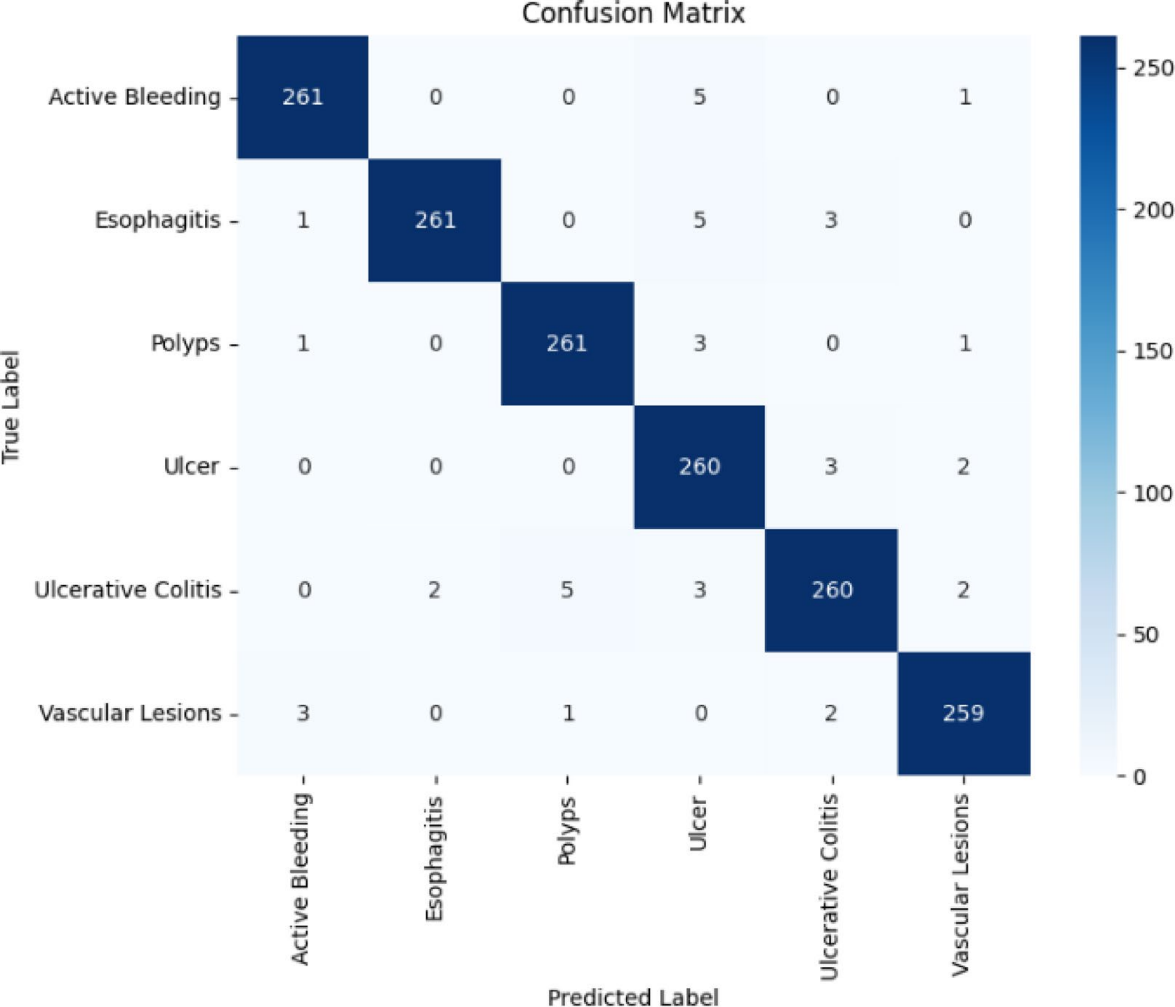
Confusion matrix of the proposed RMP-GKPS-transformer model.

The confusion matrix in Fig. 4 illustrates the performance of the model in classifying various gastrointestinal conditions, highlighting both its strengths and areas for improvement. Overall, the model demonstrated a solid ability to accurately identify most conditions, particularly Esophagitis and Ulcerative Colitis, where it achieved high correct classification rates.

An ablation study was conducted to show the effectiveness of the integration of textual features in the classification task. Table 2 provides the ablation study results comparing the performance of the proposed model with and without the inclusion of the Bio-RoBERTa model, along with the percentage improvement.

**Table 2:** Ablation study: effectiveness of Bio-RoBERTa.

Metric	Proposed model (With Bio_RoBERTa)	Without Bio_RoBERTa	Percentage improvement (%)
Accuracy (%)	99.82	96.47	3.47
Precision (%)	99.12	95.68	3.60
Recall (%)	98.84	94.35	4.76
F1 Score (%)	99.23	94.96	4.49
AUC-ROC (%)	99.3	96.2	3.22
Dice coefficient (%)	98.7	94.8	4.11
mPA (%)	98.3	94.1	4.47
IoU (%)	97.5	93.7	4.06
Execution time (ms)	122	131	6.87

The inclusion of Bio-RoBERTa in the proposed model significantly enhances performance across multiple metrics. Accuracy improves by 3.47%, as Bio-RoBERTa enables a more accurate representation of clinical notes and diagnostic observations, leading to better classification outcomes. Precision and recall see improvements of 3.60% and 4.76%, respectively, as the model effectively captures nuanced textual features, reducing both false positives and false negatives. The F1 score improves by 4.49%, demonstrating the robustness of Bio-RoBERTa in balancing precision and recall while complementing visual features. Notably, AUC-ROC and Dice Coefficient also increase by 3.22% and 4.11%, respectively, due to enhanced text embeddings that contribute to better classification boundaries and segmentation accuracy. Furthermore, while the inclusion of Bio-RoBERTa enhances accuracy and overall performance, it introduces a 6.87% increase in execution time due to the additional computational overhead of processing complex text embeddings. Despite this, the improved classification and segmentation accuracy justify the trade-off, demonstrating the effectiveness of Bio-RoBERTa in capturing nuanced textual features and complementing visual data.

Also, to validate the impact of the PPO component in the classification stage, another ablation study was conducted by replacing PPO with two alternative methods: a standard Multi-Layer Perceptron (MLP) and a softmax classifier. The results are shown in (Table 3). All other components of the proposed framework were kept constant to isolate the impact of PPO on performance.

**Table 3:** Ablation study: effectiveness of PPO in the classification Module.

Metric	Proposed (with PPO)	Replacing PPO with MLP	Replacing PPO with softmax
Accuracy (%)	99.82	98.31	97.85
Precision (%)	99.12	96.47	95.03
Recall (%)	98.84	95.66	94.11
F1 score (%)	99.23	96.06	94.57
AUC-ROC (%)	99.3	97.1	96.5

Replacing PPO with MLP results in a 1.51% drop in accuracy, with notable reductions in recall and F1 score, suggesting poorer generalization and increased misclassifications, especially in edge cases. Using a softmax layer further reduces accuracy by 1.97%, and shows the lowest F1 and AUC-ROC scores, indicating that static classifiers lack the adaptive capacity of PPO when dealing with conflicting or noisy multimodal features. The PPO-enhanced model dynamically adjusts modality importance during classification, which is critical when visual and textual inputs present contradictory or ambiguous evidence. These results demonstrate the necessity and advantage of incorporating reinforcement-based decision-making in medical multimodal classification, particularly for the high-risk tasks like GI disease diagnosis.

### Comparison analysis

The performance metrics of the proposed model is compared with those of other existing state of the arts models reviewed in the literature such as TransMSF^20^, FLATer^23^, VGG-19 + CNN^25^ and GastroNet^26^.

**Fig. 5:**
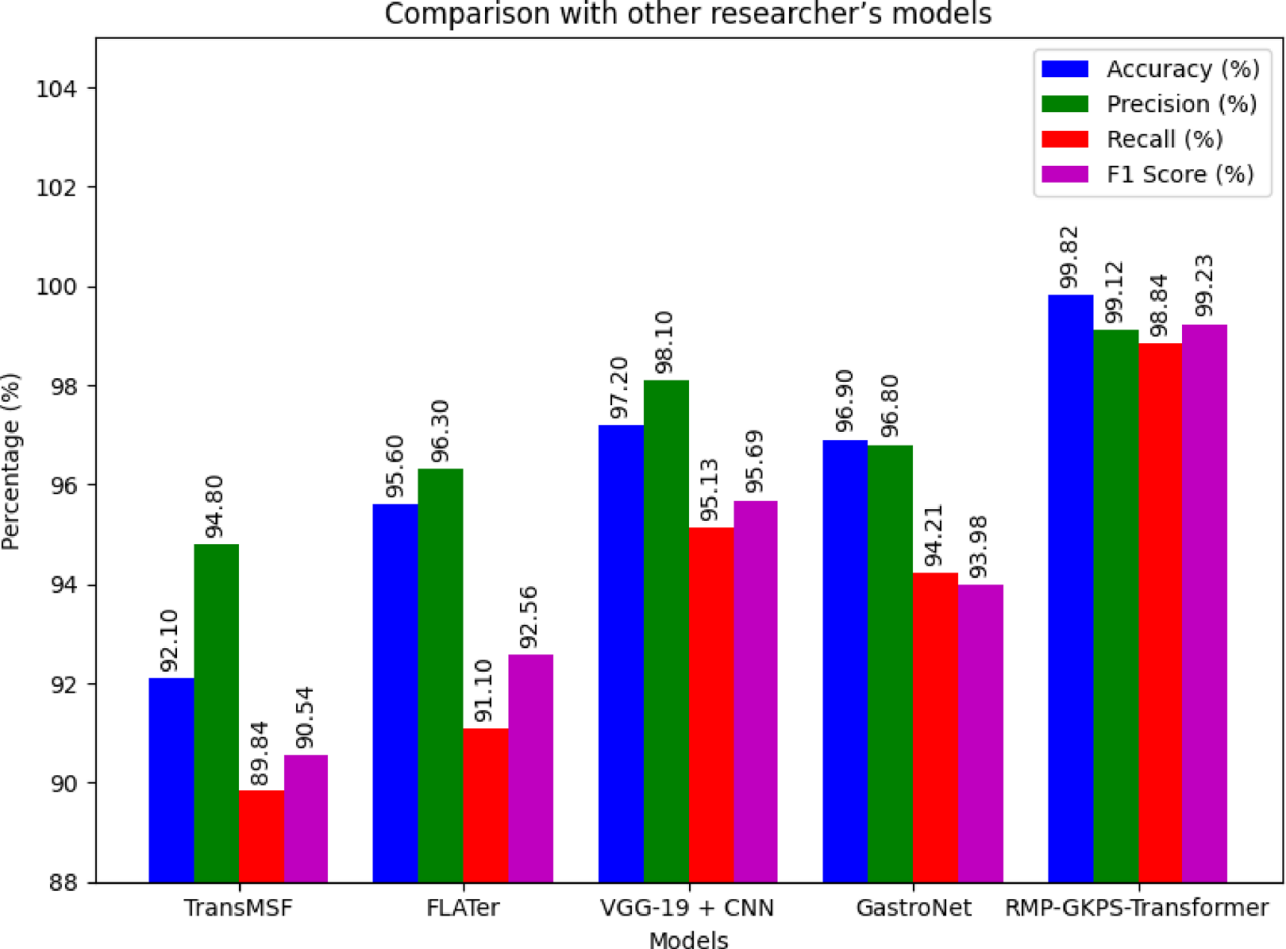
Overall comparison performance metrics of the proposed model with the existing method.

The proposed RMP-GKPS-Transformer Network outperforms state-of-the-art methods across all performance metrics as seen from (Fig. 5). Achieving an accuracy of 99.82%, the model demonstrates superior classification ability, thanks to its robust multi-modal feature integration and attention mechanisms, which minimize misclassification. With a precision of 99.12% and recall of 98.84%, the proposed approach balances the identification of true positives while avoiding false positives, attributed to the use of the Proximal Policy Optimization (PPO) and sparse RBF kernels. The F1 Score of 99.23% further highlights its ability to maintain a strong balance between precision and recall, surpassing other methods like TransMSF and FLATer, which lack the advanced hierarchical attention and recurrent optimization features of the proposed framework.

**Fig. 6:**
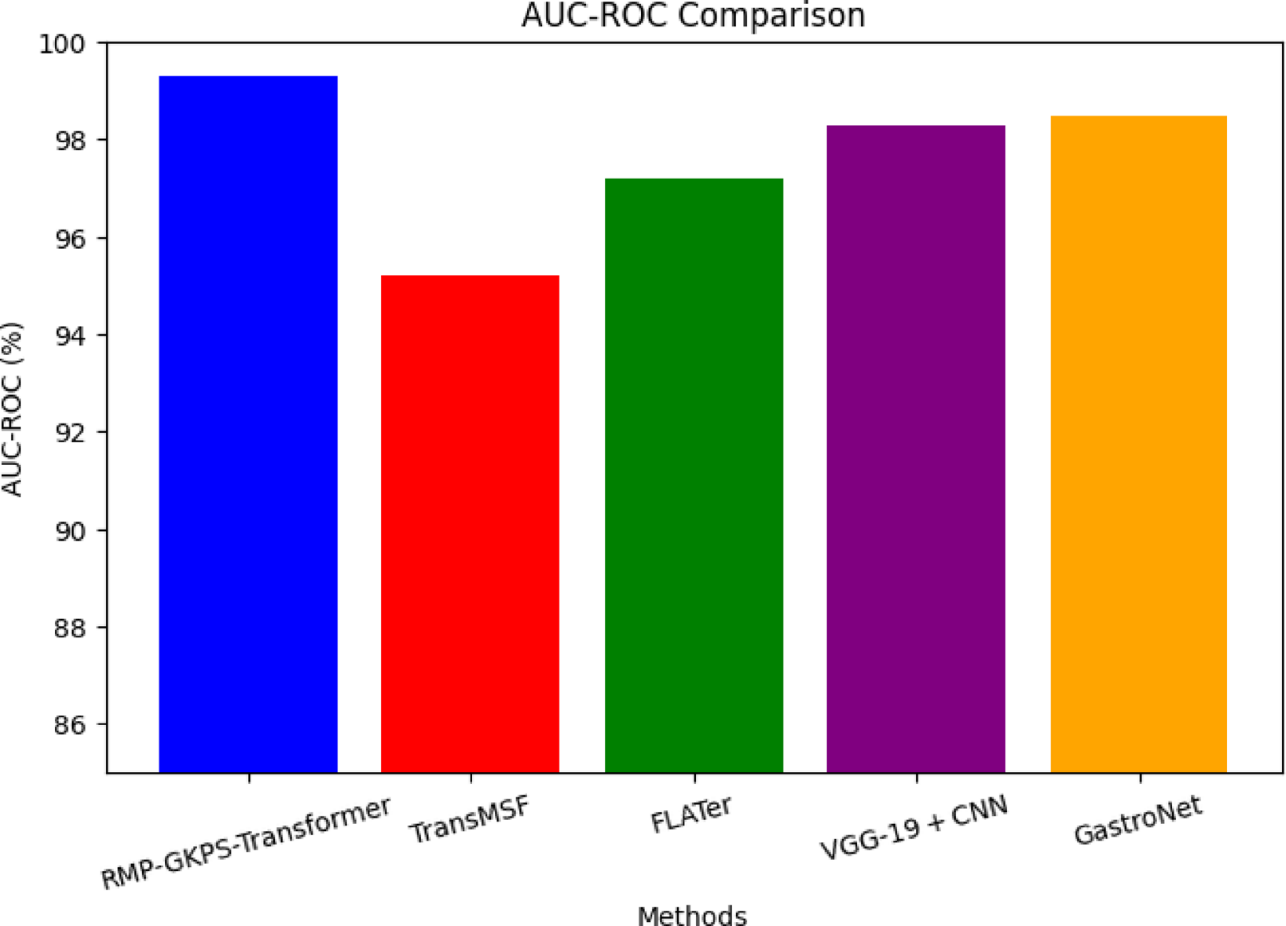
Comparison of AUC-ROC.

From Fig. 6, it is seen that proposed approach achieved the highest AUC-ROC (Area Under the Receiver Operating Characteristic Curve) of 99.3%, indicating superior performance in distinguishing between classes across all thresholds. This improvement is attributed to the integration of the Recurrent Multimodal Principal Gradient K-Proximal Sparse Transformer Network, which efficiently combines multi-modal data and captures intricate disease-specific patterns. Hence, this is surpassing the feature extraction and classification capabilities of TransMSF, FLATer, VGG-19 + CNN, and GastroNet.

**Fig. 7:**
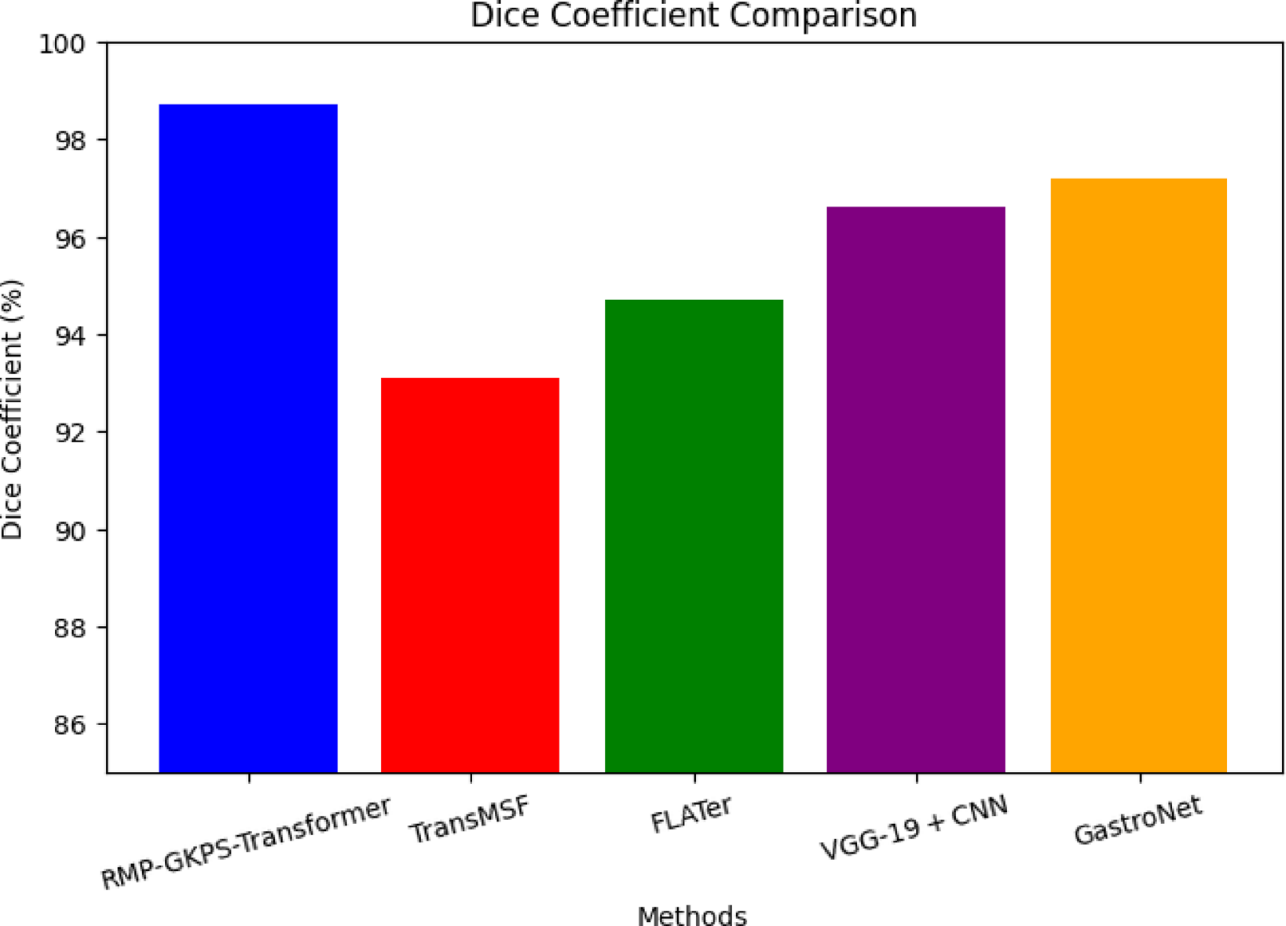
Dice coefficient.

The proposed approach achieved a dice coefficient of 98.7%, significantly higher than the other methods as shown in (Fig. 7). This indicates superior segmentation accuracy and overlap between predicted and ground truth regions. The enhancement is due to the Spatial Channel Attention mechanism and the CRF refinement stage, which ensure accurate segmentation of disease-relevant regions, outperforming the traditional feature learning methods employed by other frameworks.

**Fig. 8:**
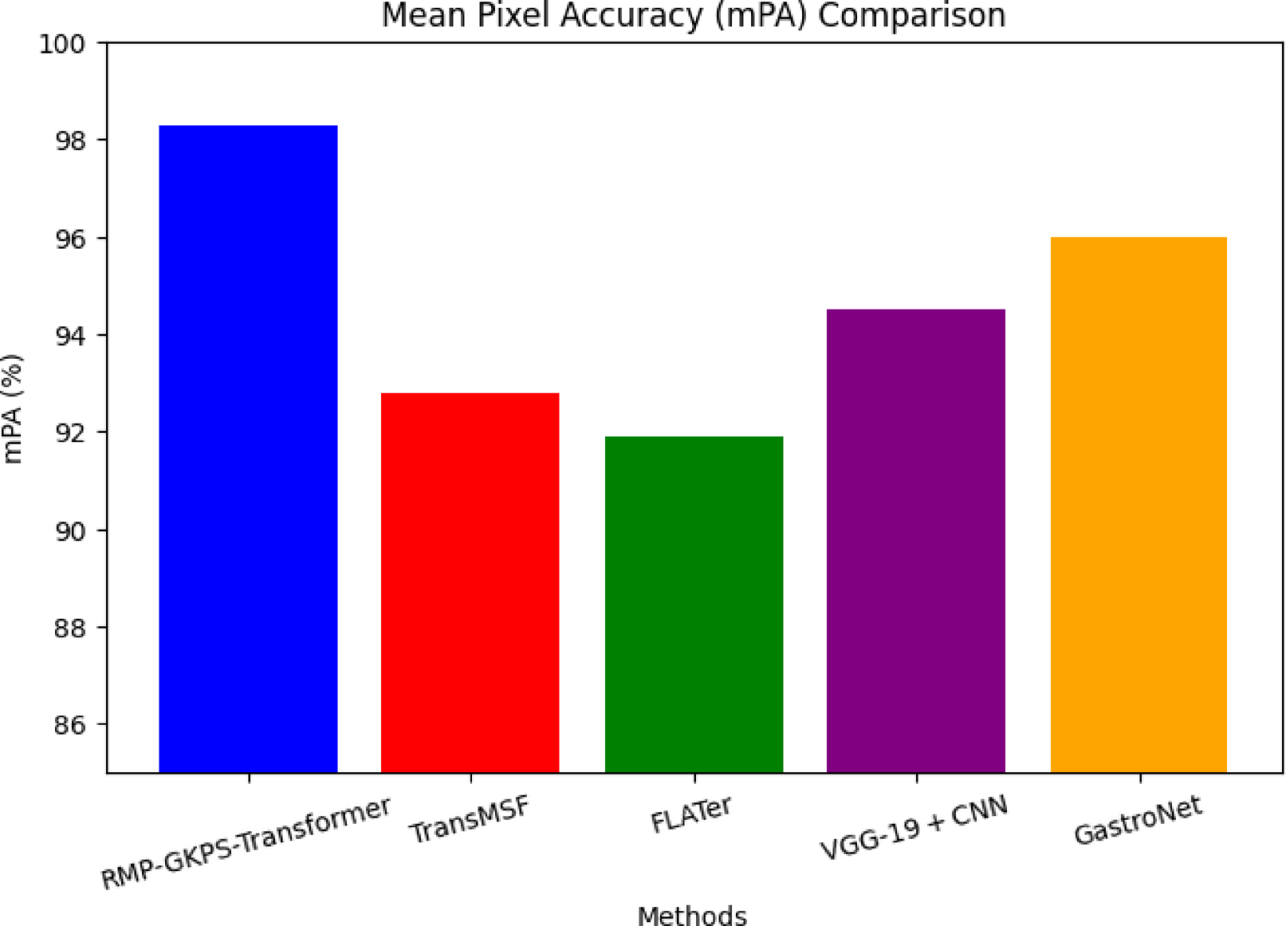
Mean pixel accuracy (mPA).

Figure 8 shows that the proposed model achieved an mPA of 98.3%, highlighting its effectiveness in pixel-level classification. The advanced ViT-based feature extraction and the hierarchical attention mechanism employed by the proposed method ensure better class-wise pixel prediction accuracy. This resolves modality conflicts more effectively compared to the suboptimal pixel-wise modeling in TransMSF, FLATer, and GastroNet.

**Fig. 9:**
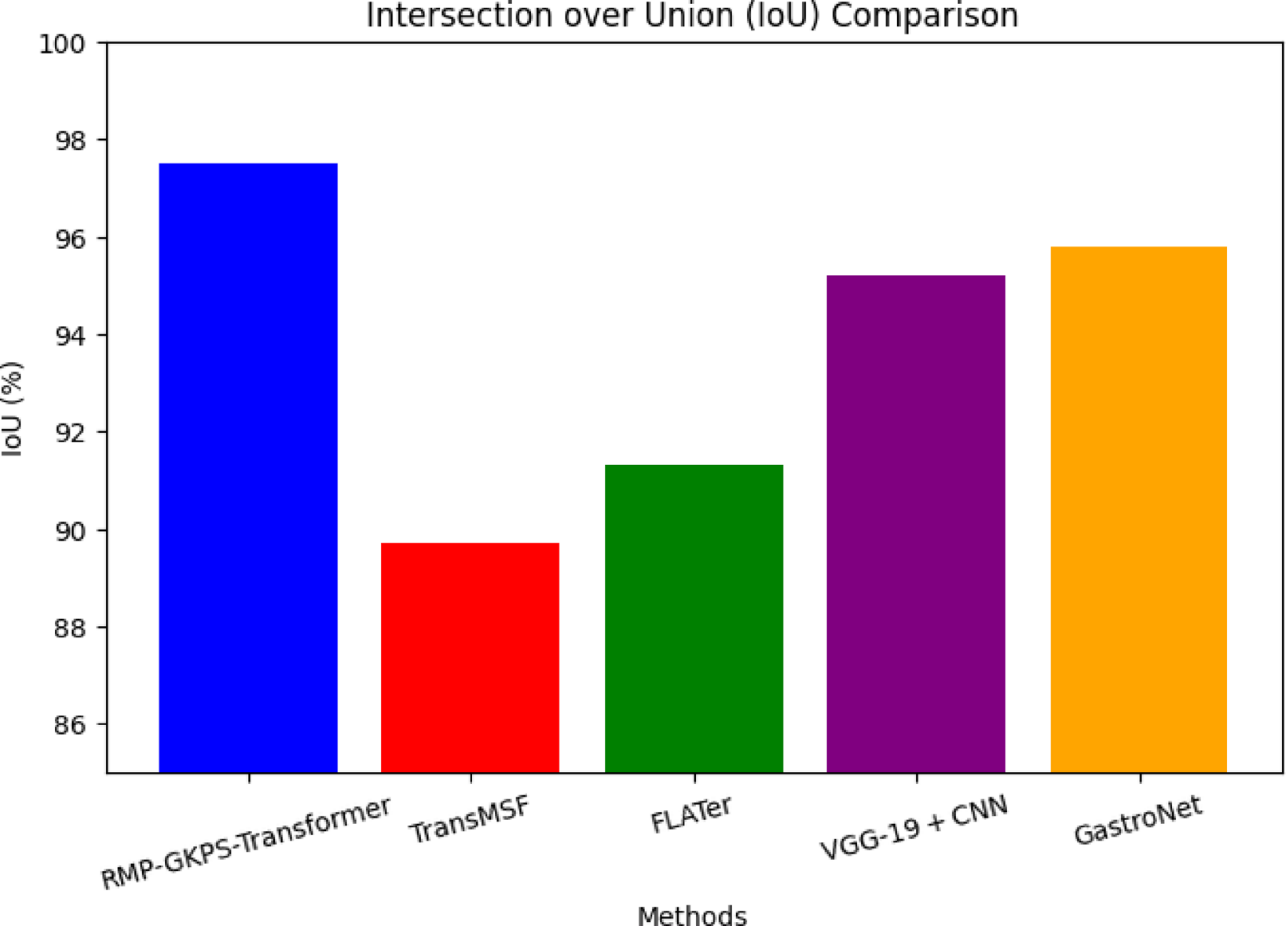
Intersection over union (IoU).

From Fig. 9, it is seen that the proposed approach demonstrates a higher capability to accurately localize disease regions with an IoU of 97.5%. The combination of sparse transformer networks and CRF-based segmentation helps mitigate redundancy while enhancing feature localization. This outperforms the IoU scores of competing methods, which rely on less refined or less integrated segmentation techniques.

**Fig. 10:**
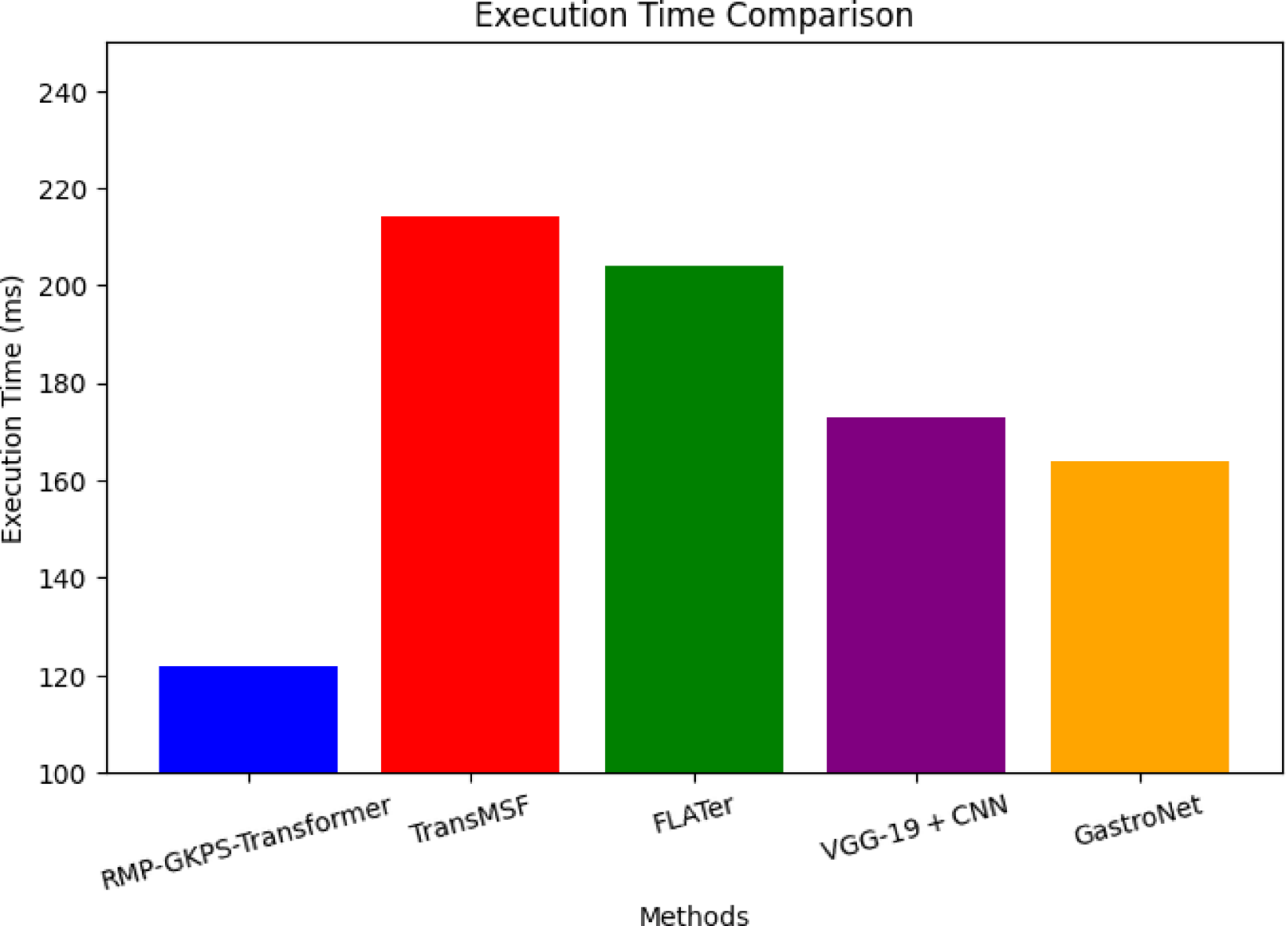
Execution time.

The proposed method achieves the lowest execution time of 122 ms, demonstrating its computational efficiency as depicted in (Fig. 10). The incorporation of sparse RBF kernels and optimized recurrent mechanisms minimizes redundant computations and enables faster processing compared to the heavier architectures of TransMSF and FLATer. The execution time advantage ensures real-time applicability, unlike the relatively slower performance of other methods.

**Table 4:** Overall comparison with other researcher’s work.

Metrics	TransMSF^20^	FLATer^23^	VGG-19 + CNN^25^	GastroNet^26^	Proposed RMP-GKPS-transformer
Accuracy (%)	92.1	95.6	97.2	96.9	99.82
Precision (%)	94.8	96.3	98.1	96.8	99.12
Recall (%)	89.84	91.1	95.13	94.21	98.84
F1 Score (%)	90.54	92.56	95.69	93.98	99.23
AUC-ROC (%)	95.2	97.2	98.3	98.5	99.3
Dice coefficient (%)	93.1	94.7	96.6	97.2	98.7
mPA (%)	92.8	91.9	94.5	96.0	98.3
IoU (%)	89.7	91.3	95.2	95.8	97.5
Execution time (ms)	214	204	173	164	122

The overall comparison Table 4 shows that the proposed model achieved the highest accuracy (99.82%), precision (99.12%), recall (98.84%), and F1 score (99.23%), indicating its robustness in correctly classifying gastrointestinal diseases while minimizing false positives and negatives. The AUC-ROC (99.3%) and Dice Coefficient (98.7%) highlight the model’s effectiveness in achieving precise classification boundaries and segmentation accuracy. The mPA (98.3%) and IoU (97.5%) values further validate its capacity for accurate pixel-level disease localization. Additionally, the proposed model significantly outperformed others in terms of execution time (122 ms), demonstrating its computational efficiency despite achieving higher accuracy. This superior performance is attributed to the integration of the RMP-GKPS-Transformer Network, effective feature prioritization, and advanced ensemble-based classification techniques, making it well-suited for real-time diagnostic applications.

The proposed RMP-GKPS-Transformer exhibits an approximate 6.87% increase in execution time compared to baseline configurations without Bio-RoBERTa. This additional time is attributed primarily to the contextual embedding generation phase, where Bio-RoBERTa processes long clinical text sequences using multi-layer transformer blocks. Despite this, the overall execution time of 122 ms remains 28.5% faster than TransMSF (214 ms) and 40.1% faster than FLATer (204 ms). This performance gain is achieved due to the Sparse Transformer’s reduced complexity of $\:O\left(n\:log\:n\right)$, which minimizes token-wise self-attention calculations during image feature encoding. Additionally, the Sparse RBF kernel and PCA-driven dimensionality reduction reduce the computational burden during classification by pruning redundant features. Therefore, the integration of Bio-RoBERTa presents a computational trade-off that yields significant gains in interpretability and diagnostic accuracy, with only a marginal increase in latency that remains acceptable for near-real-time clinical applications.

## Practical limitations, explainability, and future directions

Despite the strong performance of the proposed RMP-GKPS-Transformer framework in gastrointestinal disease diagnosis, several practical limitations and improvement opportunities remain. The architecture relies on high-capacity components such as transformer encoders and reinforcement-based classifiers (e.g., PPO), which introduce increased computational demands. This may limit its direct deployment in low-resource clinical settings or on edge devices lacking dedicated GPUs. Moreover, the current implementation uses textual data derived from publicly available abstract datasets, which may not fully represent the structure, terminology, and diagnostic nuance found in real-world clinical notes. Another critical limitation lies in the absence of longitudinal patient data, which restricts the model’s ability to track and model disease progression across time a factor highly relevant in chronic GI conditions such as ulcerative colitis. Furthermore, while the model demonstrates high performance, its decision-making process remains largely opaque to end-users, which can limit clinical trust and interpretability.

To address these limitations, future research will focus on enhancing real-time deployment feasibility through model compression techniques such as transformer pruning, quantization, and knowledge distillation. These techniques will reduce the memory footprint and computational load, enabling the framework to function effectively on embedded medical devices. Additionally, explainable AI (XAI) methods—including SHAP, Grad-CAM, and attention heatmap visualizations will be incorporated to improve model transparency and support clinician interpretation of diagnostic outputs. The generalizability of the model will be further validated using multi-institutional datasets, capturing diverse clinical scenarios, image acquisition settings, and report formats. Finally, domain adaptation strategies will be explored to enhance robustness across varying hardware, imaging protocols, and population demographics, ultimately improving the model’s utility in real-world, heterogeneous healthcare environments.

## Conclusion

In this study, a novel framework for gastrointestinal disease classification was proposed, integrating textual and visual modalities to achieve superior diagnostic accuracy. The model utilized a Recurrent Multimodal Principal Gradient K-Proximal Sparse (RMP-GKPS) Transformer Network, which combined cross-attention mechanisms, dimensionality reduction techniques, and conflict resolution strategies for robust multi-modal feature fusion. The classification stage employed an ensemble of Random Forest KNN, Proximal Policy Optimization, and Sparse Radial Basis Function kernels, ensuring balanced feature utilization, dynamic decision-making, and improved generalization. The experiments demonstrated the effectiveness of the proposed approach in classifying esophagitis, ulcerative colitis, ulcers, polyps, active bleeding, and vascular lesions, achieving state-of-the-art performance with metrics such as 99.82% accuracy, 99.12% precision, 98.84% recall, and 99.23% F1 score. Comparative analysis and ablation studies highlighted the significant contributions of Bio-RoBERTa-based text embedding and advanced fusion techniques in enhancing diagnostic performance. The system also addressed disease-specific challenges such as subtle inflammatory patterns, overlapping features, and temporal inconsistencies through innovative prioritization and alignment strategies.

## Data Availability

Supplementary data to this article can be found online at (https://www.kaggle.com/datasets/chaitanyakck/medical-text), (https://www.kaggle.com/datasets/meetnagadia/kvasir-dataset).
